# Potential Contributions of Behavior Analysis to Research on Pro-environmental Behavior

**DOI:** 10.3389/fpsyg.2022.685621

**Published:** 2022-05-17

**Authors:** Farina Wille, Florian Lange

**Affiliations:** ^1^Division of Research Methods and Biopsychology, Institute of Psychology, Technische Universität Braunschweig, Braunschweig, Germany; ^2^Behavioral Economics and Engineering Group, KU Leuven, Leuven, Belgium

**Keywords:** pro-environmental behavior, behavior analysis, context, consequences, intervention, measuring behavior

## Abstract

Large parts of contemporary research on pro-environmental behavior focus on mechanistic explanations and mental constructs. Exclusive reliance on this approach may hinder the search for novel solutions to conceptual problems, more powerful methods, and innovative behavior change interventions. Theoretical diversity, on the other hand, can render a field adaptive in its responses to crises and impasses. Against this background, we describe the complementary approach of behavior analysis and its potential contributions to problems of contemporary research on pro-environmental behavior. Behavior analysis (1) provides a consistent account of phenomena that are difficult to reconcile with the mechanistic perspective, (2) redirects the spotlight to context, (3) provides a framework and methodology for assessing behavior with actual environmental impact, and (4) could inspire the development of new intervention techniques. Based on these contributions, we conclude that behavior analysis could substantially enrich research on pro-environmental behavior.

## Introduction

Theoretical approaches guide the work of behavioral scientists (Glanz et al., 2008; van Lange, 2013). This guidance is necessarily selective: it favors some explanations, methods, and interventions at the cost of others (Crosby et al., 2002). Exclusive reliance on one theoretical approach may limit the success of a field. Theoretical diversity, on the other hand, can render a field adaptive in its responses to crises and impasses. As behavioral scientists interested in the study of pro-environmental behavior, we believe that our field could benefit from broadening its theoretical focus in its search for novel ways to address environmental issues.

In particular, we perceive contemporary research on pro-environmental behavior to be dominated by a focus on mechanistic, “social psychology-based theories” (Gifford et al., 2011, p. 442). Within this approach, researchers primarily study the role of mental states and mechanisms that are assumed to cause pro-environmental behavior. In this article, we do not wish to question the contributions of this approach, but rather highlight the contributions of an alternative approach with complementary strengths: behavior analysis.

## Principles of Behavior Analysis

Behavior analysis is a natural-science approach to understanding the behavior of individuals (APA Div. 25: Behavior Analysis, 2013; Pierce and Cheney, 2017). This means that behavior is studied as a function of natural (rather than immaterial, mental) events and processes (The Association for Behavior Analysis International, 2020). By this means, behavior analysts seek to discover the principles that guide behavior and to apply these principles to solve behavioral problems.

**Figure 1:**
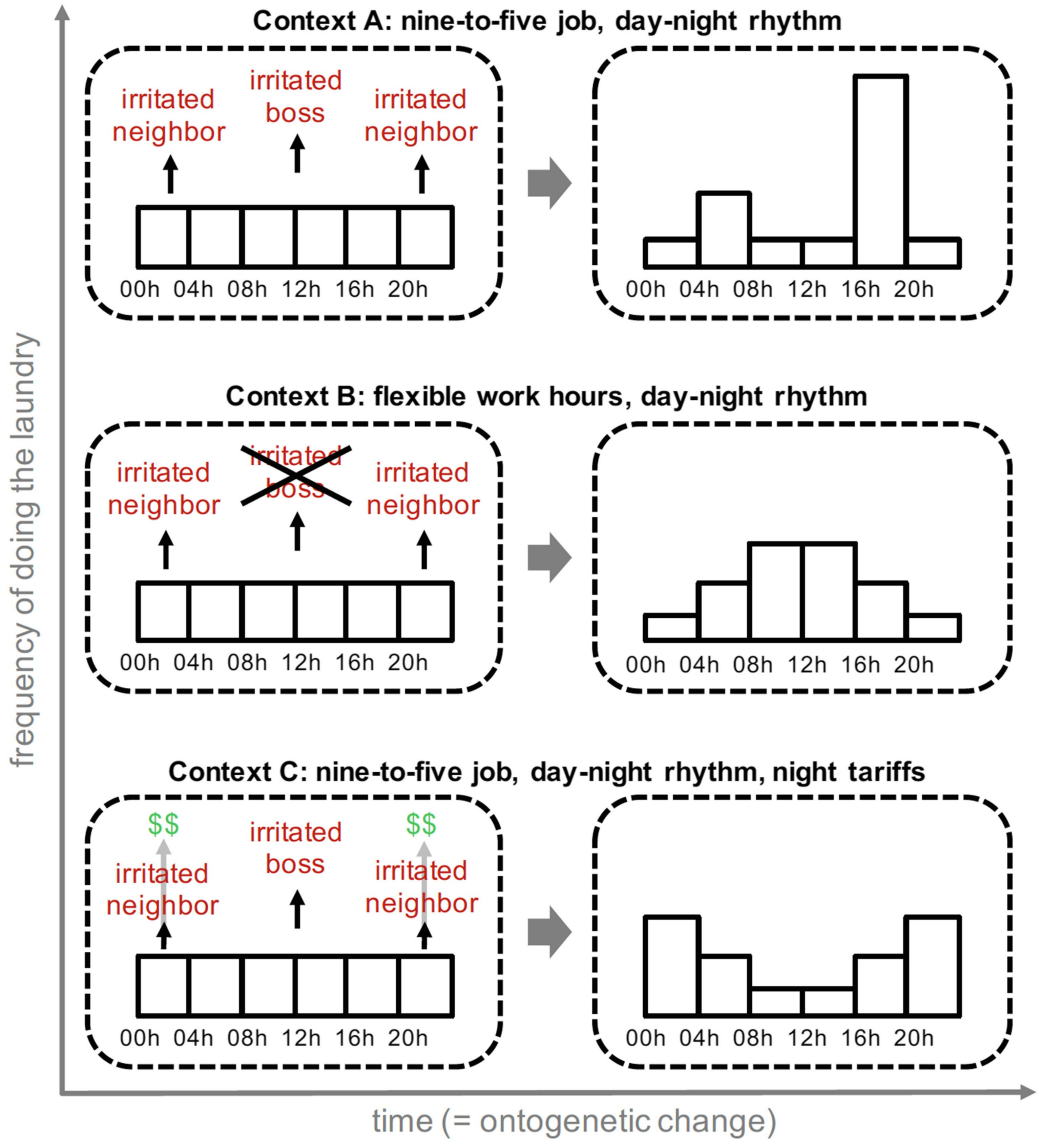
Example of ontogenetic selection for doing laundry in three different contexts. Energy using behaviors (e.g., doing laundry) produce different consequences when performed at different times of the day. Critically, these consequences depend on the context of energy using behavior (e.g., the working hours of the individual; Wille, 2021). Context-dependent consequences select the behavior and, as a result, the distribution of energy using behavior changes over time (i.e., it adapts to the context; Wille, 2021). In some contexts (e.g., Context A), this may lead to an environmentally disadvantageous behavior distribution (i.e., evening peaks of household energy consumption when energy supply from renewable sources tend to be low). In order to approach such environmental issues, the perspective of behavior analysis suggests to modify the context in a way that behavior produces different consequences (i.e., to rearrange contingencies), so that the ontogenetic development of behavior can take a different direction. In Context B, for example, a context change (i.e., the flexibilization of work hours) entails that doing the laundry at noon does not lead to negative professional consequences (e.g., falling from favor with your employer, loss of earnings). With one of the main reasons for the evening peak in energy consumption being removed, behavior may become more evenly distributed across the day. Similarly, Context C involves a differential tariff structure that rewards doing the laundry at night. Implementation of such tariff structures can change how energy using behavior is selected and thus distributed across the day. It is noteworthy however that such tariff structures are unlikely to be as effective as suggested in this figure because of the dominating influence of other elements of the existing context (e.g., inflexible working hours; Wille, 2021).

Based on the work of Skinner (1953, 1974), behavior analysis is characterized by a focus on *contingencies* (i.e., the relationships between environment, behavior, and its consequences) as a key concept in behavioral explanation. It proceeds from the observation that behavior, in interaction with the environment, produces consequences in the physical world. For example, cycling to work on a rainy day may produce the consequence of being soaked and wearing a fur coat at an animal welfare rally may produce the consequence of raised eyebrows. Contingencies are assumed to select the behavior of individuals and it is this selection which is the central mode of causation in behavior analysis. Notably, it parallels the mode of causation in natural selection (Skinner, 1981, see also Glenn, 1988; McDowell, 2004, 2019; Baum, 2017; see Borgstede and Eggert, 2021 for a unified account), but in contrast to natural selection, selection does not occur across generations, but across situations within the lifetime of the individual (i.e., ontogenetically, see Figure 1 for an example).

The empirical program of behavior analysis reaches from laboratory experiments with non-human animals to field studies in societally relevant contexts. The experimental analysis of behavior, on the one hand, is concerned with examining the effects of systematically manipulated contingencies under controlled circumstances. The data obtained from this analysis are then inductively integrated into principles of behavior. Applied behavior analysts, on the other hand, make use of the principles discovered in the experimental analysis of behavior to address behavioral problems in less controlled circumstances. For example, they may account for pro-environmental behavior by referring to principles of operant conditioning, delay-discounting or generalization (Lemos et al., 2019; Schneider and Sanguinetti, 2021; Wille, 2021). Based on a functional analysis of the contingencies that maintain a behavior, applied behavior analysts aim to rearrange contingencies to promote alternative behaviors. The effect of these rearrangements is typically studied longitudinally, for example, through introducing, removing, and reintroducing an intervention (Bailey and Burch, 2018). By this means, it is possible to demonstrate that a target behavior (e.g., gasoline consumption) of a target population (e.g., car drivers in Texas during the oil crisis) varies as a function of the intervention (e.g., presenting feedback about gasoline consumption on the evening news; Rothstein, 1980).

With its focus on contingencies and experimentation, behavior analysis invites questioning of existing societal incentive structures. This is nicely illustrated in *Walden Two*, a novel written shortly after World War II (Skinner, 1948). In Walden Two, Skinner describes an egalitarian community of close to a thousand members who are encouraged “to view every habit and custom with an eye to possible improvement” (1948, p. 25). Practices, policies, and community structures are subject to continuous experimentation and selected based on evidence rather than dogmatism. From this approach, context changes emerge that are found to promote the sustainability of the community and the well-being of its members. These experimental practices reflect Skinner’s intention to present Walden Two as an illustration of how behavior analysis can contribute to a sufficiency-oriented alternative to consumerism (and the associated environmental pollution; Skinner, 1976). Issues of environmental sustainability are treated to an extent that may be considered atypical for the 1940s (Altus and Morris, 2009). Members of Walden Two build energy-efficient buildings, practice sustainable agriculture, reduce food waste, and share their facilities and devices. Nine-to-five routines are replaced with flexible, staggered schedules that allow making more efficient use of space and equipment (and to reduce crowds) and unnecessary possessions are largely avoided.

Of note, the experimental approach of Walden Two (and applied behavior analysis in general) bears close resemblance to contemporary concepts of real-world laboratories and living labs (Schäpke et al., 2018; Wanner et al., 2018), suggesting that behavior analysis may inform the search for behavioral sustainability solutions. Important tenets of behavior analysis, such as the theory of reinforcement learning, have already been incorporated successfully into other fields such as neuroscience (e.g., Schaal, 2013), behavioral economics (e.g., Rachlin et al., 1976) or neuroeconomics (e.g., Sawe and Chawala, 2021). Here we would like to specifically point to the potentials of behavior analysis that we see for the field of environmental psychology.

## Differences From Mechanistic Approaches to Studying Pro-Environmental Behavior

While the current theoretical landscape in pro-environmental behavior research is far from homogenous (Vining and Ebreo, 2002; Steg and Vlek, 2009; Kaiser et al., 2010; Bamberg, 2013; Klöckner, 2013; Gifford, 2014), it appears that most contemporary attempts to explain pro-environmental behavior do so by referring to mental constructs (e.g., attitudes, intentions, beliefs, goals). Such constructs, internal to the individual, are seen as proximate, *mechanistic* causes of behavior. Behavior analysis employs a different mode of causation for the explanation of behavior. Behavior analysts seek to describe orderly relationships between context, behavior and its consequences, and they refer to these *functional* relationships to explain how a behavior has been selected over the lifetime of an organism (see also Skinner, 1985; Hineline, 1990; Todd and Morris, 1992; Chiesa, 1994; Moore, 1996; Moore, 2003; Leigland, 2010). In doing so, they do not deny the existence of mechanistic causes or mental constructs, they are simply interested in another type of explanation (i.e., in another one of Tinbergen’s four questions; Tinbergen, 1963).

**Figure 2:**
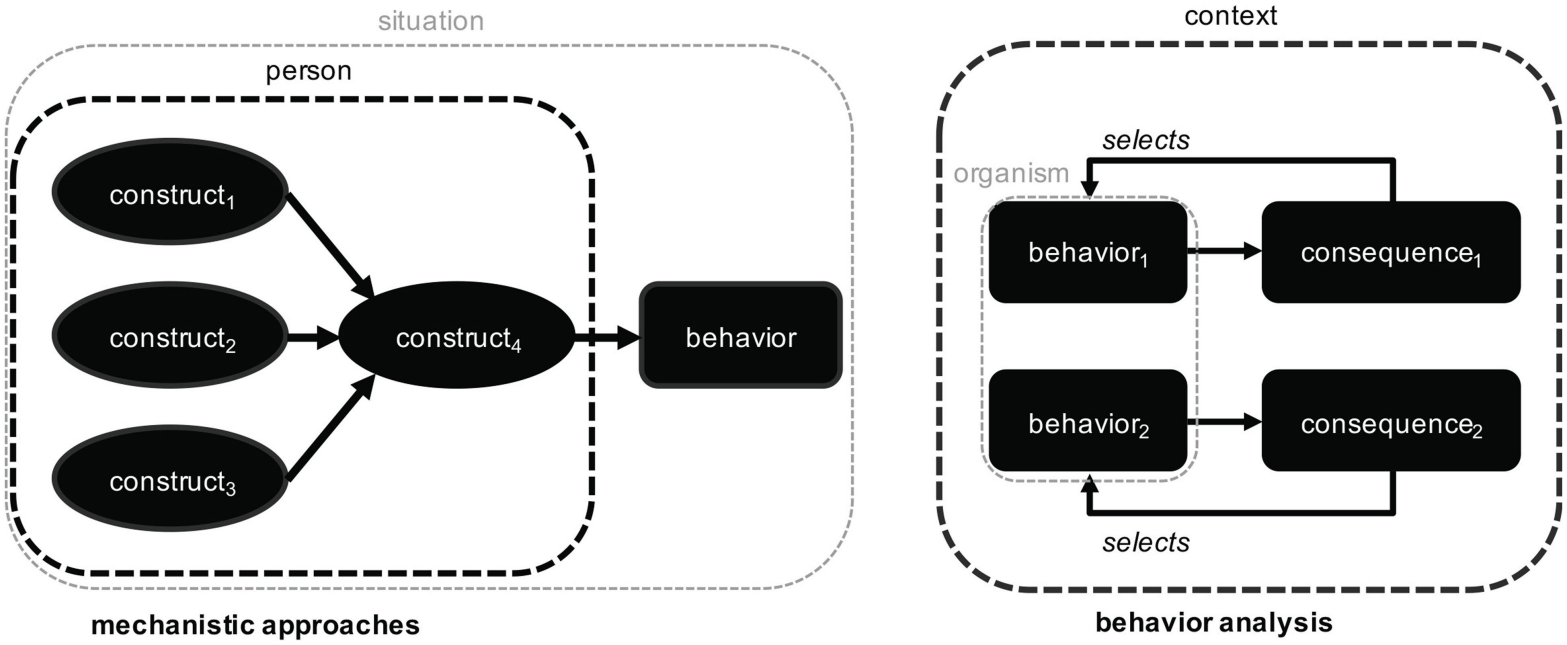
Comparison between mechanistic approaches and behavior analysis. Mechanistic approaches to explaining pro-environmental behavior (on the left, with a focus on mental processes within an individual) is contrasted with behavior analysis (on the right, with a focus on behavior-consequence relationships in context).

As a corollary, behavior analysis and mechanistic approaches differ in the role and importance they assign to context factors in explaining behavior (Figure 2). Behavior analysts consider contextual characteristics to be relevant to the degree to which they determine the consequences of behavior, whereas in mechanistic approaches, context factors are considered relevant if they affect mental mechanisms. When trying to explain pro-environmental behavior, researchers working within mechanistic approaches would rather focus on identifying mental constructs that explain a meaningful amount of variance in that behavior. Behavior analysts, by contrast, would aim to identify contextual contingencies that maintain or alter the behavior in question. They might also take into account potential interrelations between different behaviors. For example, a behavior analysis account of participating in pro-environmental demonstrations might not only refer to the consequences that accompany participation, but also to the consequences that maintain other behaviors (e.g., going to the gym) when they conflict with participation.

These theoretical differences also affect the type of methodology researchers use when studying pro-environmental behavior. On the one hand, the focus on mental constructs relates to the development of assessment tools (e.g., self-report scales) that are assumed to provide information about those constructs. These tools are then used to study the relationship between mental constructs and pro-environmental behavior, often in correlational survey studies. On the other hand, the focus of behavior analysis on behavior in its own right comes with an emphasis on field observational and experimental studies that examine behavior as a function of changing environmental contingencies.

## Discussion: Potential Contributions to Research on Pro-Environmental Behavior

In our view, the abovementioned theoretical and methodological differences point to the great potential of embracing behavior analysis as a complementary approach to studying pro-environmental behavior. With its focus on determinants and explanations that receive less attention within mechanistic approaches, behavior analysis can enrich the search for behavioral solutions to sustainability problems. This is neither speculation nor a new idea. In fact, behavior analysis has a longstanding tradition in applying its principles to the study and promotion of pro-environmental behavior research (Cone and Hayes, 1980; Dwyer et al., 1993; Lehman and Geller, 2004; Foxall et al., 2006; Gelino et al., 2021; Schneider and Sanguinetti, 2021). However, only few behavior analysts currently work in the field of environmental psychology (Schneider and Sanguinetti, 2021). As a consequence, we suspect that many environmental psychologists might be unaware of the potential contributions of behavior analysis to the study of pro-environmental behavior.

### Behavior Analysis Could Help Resolve Inconsistencies of Pro-environmental Behavior Research

A different theoretical perspective can shed new light on longstanding problems within a dominant scientific paradigm. One such problem within mechanistic approaches to explaining (pro-environmental) behavior is the so-called attitude-behavior gap (LaPiere, 1934; Kollmuss and Agyeman, 2002; Glasman and Albarracín, 2006; see Carrus et al., 2021, for a recent meta-analysis in the domain of energy saving). Observed inconsistency between pro-environmental behavior and attitudes towards this behavior (as assessed *via* verbal statements) may seem puzzling when the former is assumed to causally depend on the latter. As a consequence, numerous attempts have been made to address this inconsistency within mechanistic approaches. In contrast, this inconsistency appears unproblematic and irrelevant from a behavior analysis perspective. Behavior analysts would not expect two different behaviors such as verbal attitudinal statements and overt pro-environmental behavior to converge (see also DeFleur and Westie, 1963). In most cases, such behaviors can be expected to diverge because they are the result of different selecting contingencies. For example, verbal statements about buying environmentally friendly laundry detergents may be a function of consequences such as positive verbal affirmations of peers, while picking up a laundry detergent from the supermarket shelf might be a function of consequences such as a higher monetary loss in comparison to another laundry detergent.

This example illustrates how a different theoretical perspective such as behavior analysis could help identify conceptual impasses and refocus research priorities. Instead of investing a lot of resources closing a putatively problematic attitude-behavior gap, behavior analysts would separately study the determinants of overt pro-environmental behavior and the determinants of verbal behavior about the attitude object. Both behaviors can be of theoretical or practical importance, but they need not be the same or causally linked to each other.

### Behavior Analysis Could Promote the Study of Context

If context factors are selected for scientific analysis primarily based on their assumed relevance for mental constructs and mechanistic explanations of pro-environmental behavior, important contextual determinants may be overlooked (see also Nielsen et al., 2021). With its theoretical focus on functional relationships and contingencies, behavior analysis may promote a more comprehensive and systematic selection and study of contextual determinants. To explain a behavior, behavior analysts examine how the behavior modifies the context and what consequences this context modification has on the behavior of the individual. They would observe, for example, that a student’s social context undergoes considerable changes after the student has switched to a vegetarian diet. The student might spend more time with some people and less time with others, receive encouragement from some friends and skeptical comments from others. In turn, these consequences might affect the student’s behavior. The student might give up on dairy products as well, return to eating meat, or develop variations in eating behavior dependent on the context of the meal. Functional context-behavior relationships of that kind are described within the framework of behavior analysis and they can offer a powerful means to clarify the effects of contextual factors on pro-environmental behavior.

Thinking about contextual factors in terms of the consequences they produce is likely to enlarge the set of factors that researchers explore as potential determinants of pro-environmental behavior. It may also generate more practically relevant insights on how the environment needs to be designed to facilitate pro-environmental behavior. While correlations between pro-environmental behavior and its perceived difficulty (e.g., Fujii, 2006) do not tell us how perceived difficulty (and thus behavior) can be changed, finding the rate of recycling behavior to vary as a function of the independently manipulated distance to recycling facilities (O’Connor et al., 2010) provides directly applicable behavior-change knowledge.

### Behavior Analysis Could Promote the Measurement of Actual Behavior

Most studies in contemporary pro-environmental behavior research rely on self-report measures (Lange et al., 2018) that face a variety of validity problems (Gifford, 2014; Kormos and Gifford, 2014; Lange and Dewitte, 2019; see also Hausman, 2012, for a related perspective from environmental economics). From a behavior analysis perspective, it does not make much sense to measure actual engagement in pro-environmental behavior by asking participants how they typically behave or how they would behave in a hypothetical scenario. Just as attitudinal statements, such verbal behaviors are often selected by other consequences than actual pro-environmental behavior. In consequence, applied behavior analysts have developed an alternative assessment tradition. They have relied on objective observations of actual behavior in the field, which has resulted in a rich research literature that can be informative for pro-environmental behavior researchers independent of their theoretical background. In addition to instructive analyses of methodological and conceptual aspects of behavioral assessment (Kazdin, 1979, 1982; Nelson and Hayes, 1979; Bailey and Burch, 2018), this literature contains numerous empirical examples illustrating how pro-environmental behaviors can be studied without self-reports (see Lehman and Geller, 2004; Gelino et al., 2021; for review). For example, Mayer and Geller (1982–1983) report a study involving the unobtrusive observation of cycling behavior as a function of an incentive intervention. Similarly, Geller et al. (1973) installed observers in the checkout area of a supermarket to record whether customers bought returnable versus single-use drink containers.

When it is not possible to observe behavior directly or to do so in an unobtrusive way, behavior analysts have observed the products of pro-environmental behavior. An example of this approach is provided by Foxx and Hake (1977) and Hake and Zane (1981) who recorded participants’ odometers to calculate the distance traveled by car. Along similar lines, Winett and Nietzel (1975) relied on trained undergraduate students to obtain objective readings from participants’ electricity meters and Keller (1991) has counted the number of households that placed recycling bins on the sidewalks of experimental versus control roads.

Sometimes, it might also prove beneficial to artificially arrange situations in a way that promotes experimental validity (Kazdin, 1979). For example, by actively distributing handbills in a grocery shop, Geller et al. (1977) ensured that all customers had similar opportunities to perform the behavior of interest (i.e., littering). This approach of observing behavior in contrived situations may be particularly helpful when baseline frequencies of a pro-environmental behavior are low. A special case of such contrived situations can be found in the laboratory where experimenters can exert more control over the behavior of interest. Consequential laboratory tasks have been used to study, for example, the effect of feedback (Camargo and Haydu, 2016) or contextual manipulations (Lange et al., 2020) on pro-environmental behavior. Such tasks may also help to integrate research in behavior analysis, environmental psychology, and experimental economics (Berger and Wyss, 2021).

### Behavior Analysis Could Promote the Development of (Novel) Intervention Approaches

The theoretical framework used to explain pro-environmental behavior also constrains the search for effective behavior change techniques. While researchers within a mechanistic tradition predominantly focus on intervention approaches that may alter mental constructs, behavior analysts rather target behavioral contingencies (an approach related to the concept of nudging, e.g., Tagliabue and Sandaker, 2019). Popular intervention techniques such as feedback, use of discriminative stimuli, self-monitoring, and modeling of pro-environmental behavior involve the highlighting of natural contingencies (Winett et al., 1979, 1982). In addition, behavior analysts have examined possible ways to modify contingencies. For example, participants have received cash payments contingent on reductions of their car driving activities (Hake and Zane, 1981) or electricity consumption (Winett and Nietzel, 1975). A recent meta-analysis has found such reward-based interventions to be generally effective, both during the intervention and after reward removal (Maki et al., 2016).

Critically, behavior analysis can offer more than the notion of a general reward effect (Schneider and Sanguinetti, 2021) or the mere idea of studying situational effects. For example, extensive research in the experimental analysis of behavior has focused on the effects of different reward characteristics and contingencies (Ferster and Skinner, 1957). Rewards have been found to produce more stable rates of behavior when they are given only occasionally (rather than after every performance of the desired behavior; Jenkins and Stanley, 1950). Moreover, the stability of behavior change has been shown to increase with increasing behavioral demands to be satisfied before rewards are given (Boren, 1961; Hearst, 1961). Such findings should be particularly interesting for applied pro-environmental behavior research as they indicate that more desired behavior change could be obtained with fewer rewards (thus requiring smaller financial investments; Cone and Hayes, 1980; Schneider and Sanguinetti, 2021). However, a systematic analysis of reward schedules, rates, sizes, and types in the domain of pro-environmental behavior is still lacking. Many principles and functional relations identified in the experimental analysis of behavior have been overlooked in pro-environmental behavior research (Schneider and Sanguinetti, 2021) and may contribute to the development of more (cost-)effective interventions to promote pro-environmental behavior.

## Critical Reflection and Conclusion

With its focus on different modes of explanation, different determinants of behavior, and different methodological approaches, behavior analysis can substantially enrich research on pro-environmental behavior. Behavior analysis can help redirect the focus from mental constructs to behaviors of actual environmental relevance and promote the systematic analysis of the context factors determining pro-environmental behavior. It can offer new (or neglected) concepts for changing behavior (e.g., schedules of reinforcement), powerful methods to investigate the effectiveness of interventions (e.g., paradigms for observing actual behavior), and inspiration for the societal transformation towards sustainability (e.g., Walden Two). Of course, these contributions do not uniquely follow from behavior analysis nor are they exclusively realized within behavior analysis. Researchers from other backgrounds also study actual pro-environmental behavior as a function of situational variations and altered contingencies (see, e.g., Osbaldiston and Schott, 2012; McKenzie-Mohr and Schultz, 2014; Karlin et al., 2015; Byerly et al., 2018; Grilli and Curtis, 2021; for reviews) and we do not wish to imply that environmental psychologists would need to convert to behavior analysis in order to do meaningful research. However, we do think that behavior analysis offers a consistent theory, rich research tradition, and source of inspiration that can serve to inform and further improve contemporary pro-environmental behavior research. As such, we believe that assigning a more prominent role to behavior analysis can promote the success of the field and the search for behavioral solutions to environmental issues.

Despite this potential, we acknowledge that many researchers in the field may be hesitant to draw from a perspective that has been criticized as heavily as behavior analysis and its philosophical foundation (i.e., Skinner’s radical behaviorism). Some of the most common concerns against Skinner’s radical behaviorism are that it would (1) ignore internal constructs such as consciousness and feelings (2) neglect biological and genetic differences and argue that all behavior is acquired during the lifetime of an individual (3) ignore cognitive processes (4) have no place for intention or purpose (5) have a simplistic view on language and (6) be unable to explain complex behavior (Skinner, 1974; Todd and Morris, 1983). Of note, all these points have been identified as misconceptions, they have been addressed and clarified multiple times, but nonetheless remain part of scientific debate, educational textbooks and university students’ perceptions (e.g., Skinner, 1974; Bijou, 1979; Todd and Morris, 1983, 1992; Morris, 1985; Lamal, 1995; Adelman, 2007; Arntzen et al., 2010; Racine, 2021). We hope that by presenting important behavior analysis principles in the section Principles of Behavior Analysis, we were able to disperse reservations as long as they belong to the realm of misconceptions.

In contrast, the costs of research in behavior analysis may be considered a true limitation of the approach. Observing actual behavior as a function of actual contextual changes is necessarily more expensive and time-demanding than research relying on self-report questionnaires and hypothetical scenarios. However, we believe that the benefits of this approach in terms of scientific utility and validity can be argued to justify these costs. In addition, behavior analysis (just as mechanistic approaches) does not offer a fully comprehensive explanation of behavior on all levels of analysis. Focusing on functional relationships and ontogenetic selection, behavior analysis remains silent for example about the precise intraindividual physiological mechanisms that give rise to a particular behavior in a particular moment. A fully comprehensive explanation of behavior will require integration of functional and mechanistic accounts. Such integration is a difficult endeavor [see Hineline (1990); Hughes et al. (2016) for discussion] and beyond the scope of this introductory perspective article. We hope that by presenting the functional approach of behavior analysis here, we can contribute to this integration and further discussions of its complementary merits and limitations.

## Author Contributions

All authors listed have made a substantial, direct, and intellectual contribution to the work and approved it for publication. Both authors have contributed equally to this work.

## Funding

FL received funding from the FWO and European Union’s Horizon 2020 research and innovation programme under the Marie Skłodowska-Curie grant agreement No 665501 and a FWO postdoctoral fellowship (No 12U1221N).

## Conflict of Interest

The authors declare that the research was conducted in the absence of any commercial or financial relationships that could be construed as a potential conflict of interest.

## Publisher’s Note

All claims expressed in this article are solely those of the authors and do not necessarily represent those of their affiliated organizations, or those of the publisher, the editors and the reviewers. Any product that may be evaluated in this article, or claim that may be made by its manufacturer, is not guaranteed or endorsed by the publisher.
